# The Infants' Hospital, Denning Road, Hampstead

**Published:** 1904-05-21

**Authors:** 


### THE INFANTS' HOSPITAL, DENNIN[illegible] ROAD, HAMPSTEAD.

This hospital is simply a terrace house, in no [illegible] specially well adapted for the purposes of a hospital. [illegible] are two wards, one of which contains nine cots an[illegible] other eleven. As the one leads into the other there [illegible] always be through ventilation, although at the time [illegible] visit the door between the two was closed, as there [illegible] been an outbreak of chicken-pox in the hospital an[illegible] children in the smaller ward had not been pronounce[illegible]

from infection. The only ground belonging to the hospital is a small back-yard, where, when we visited the hospital, three children were lying in cradles, getting the benefit of the air. Although the hospital has so little ground the back is fairly open, as it consists of the gardens of two rows of houses. Moreover, Hampstead Heath is within a few minutes' walk and the children are taken there daily when the weather permits. The wards are clean and dainty, and the arrangements of the hospital as good as the nature of the building permits.

The hospital was founded a year ago, and is intended solely for the reception and treatment of children from one week to two years old, who are suffering from the disorders of malnutrition. Patients are not received when the malnutrition is caused or complicated by other ailments—tuberculosis, syphilis, etc., although occasionally such cases have been accepted by mistake. The hospital is unendowed, and is entirely free—subscribers receive no letters of recommendation or other advantage in return for their donations. Patients are brought from all parts of London and the neighbourhood. Some are sent from the out-patient departments of the general hospitals, others are recommended by doctors, and others are brought by the advice of some mother whose child has already been treated there. The children are fed on "modified milk," prepared according to the Walker-Gordon method. The milk is prepared at the Walker-Gordon farm at Sudbury, and sent to the hospital daily. The diet for each patient is prescribed by the honorary physician, Dr. Ralph Vincent, and so far as the milk is concerned, is prepared according to the prescribed quantity and strength, at the farm. Each child has a box labelled with its name. This box contains a number of compartments round a central one, which contains ice. In the outer compartments the bottles for the various meals are placed, so that when a child requires feeding, the nurse has only to go to its box, which is kept in a cool cellar, take out the bottle intended for that meal, warm the milk by placing the bottle in a vessel of hot water, then put the nipple on it, and give it to the child. The risk of giving a child a wrong diet is thus reduced to a minimum. This milk practically constitutes the entire food of the patients, with a little orange juice added when necessary, and, in rare instances, an occasional dose of simple medicine. The children are weighed periodically, and the weight, the quantity, and constituents of the diets, the characteristics of the dejections, and any other details are carefully set down. The records of the cases are well kept, full and clear. The average stay of each patient is two months. No out-patient work is undertaken, but when children are removed, the matron tries to keep in touch with them by bringing each case under the notice of the district nurse, clergyman, district visitor, or any other person likely to take an interest in it in the neighbourhood in which the child lives.

The honorary physician visits the hospital twice a week, and it is when he pays these visits that patients are received. Dr. A. H. Cook, of Rosslyn Hill, Hampstead, visits in cases of emergency, and also attends the staff. The staff consists of a matron, three sisters—all fully trained—and five probationers. The probationers are at present all young women, who are thus putting in their time until they are old enough to enter a general hospital.

The expenditure is about £1,000 a year. The situation, though healthy, is inconveniently remote both for the doctor and for the parents of the patients, and, as we have said, the building is not particularly well adapted for the purpose of a hospital. On the other hand, the proximity to Hampstead Heath must be advantageous to the health of the children, and it would be difficult to get a building in a central locality equally favourably situated in this respect.

